# Esophageal abnormalities and the risk for gastroesophageal cancers—a histopathology-register-based study in Sweden

**DOI:** 10.1007/s10654-021-00833-6

**Published:** 2022-01-03

**Authors:** Isabella Ekheden, Jonas F. Ludvigsson, Li Yin, Peter Elbe, Weimin Ye

**Affiliations:** 1 Department of Medical Epidemiology and Biostatistics, Karolinska Institutet, Box 281, 171 77 Stockholm, Sweden; 2 Department of Paediatrics, Örebro University Hospital, Örebro, Sweden; 3 Division of Epidemiology and Public Health, School of Medicine, University of Nottingham, Nottingham, UK; 4 Department of Medicine, Columbia University College of Physicians and Surgeons, New York, NY USA; 5 Department for Digestive Diseases, Karolinska University Hospital, Stockholm, Sweden; 6 Division of Surgery, Department of Clinical Intervention and Technology (CLINTEC), Karolinska Institutet, Stockholm, Sweden; 7 Department of Epidemiology and Health Statistics and Key Laboratory of Ministry of Education for Gastrointestinal Cancer, Fujian Medical University, Fuzhou, China

**Keywords:** Barrett’s esophagus, Cohort study, Esophageal adenocarcinoma, Precursor lesions

## Abstract

**Background:**

The poor survival of patients with gastroesophageal cancers may improve if additional esophageal precursor lesions to Barrett’s esophagus and squamous dysplasia are identified. We estimated the risk for gastroesophageal cancers among patients with various histopathological abnormalities in the esophagus, including Barrett’s esophagus, subdivided by histopathological types.

**Methods:**

Histopathology data from esophageal biopsies obtained 1979–2014 were linked with several national population-based registers in Sweden. Patients were followed from 2 years after the first biopsy date until cancer, death, emigration, esophagectomy/gastrectomy or end of follow-up, 31st of December 2016, whichever came first. We estimated standardized incidence ratios (SIRs) as measures of relative risk with the Swedish general population as reference.

**Results:**

In total 367 esophageal adenocarcinoma (EAC) cases were ascertained during 831,394 person-years of follow-up. The incidence rate (IR) for EAC was 0.1 per 1000 person-years for normal morphology, 0.2–0.5 for inflammatory changes, and 0.8–2.9 for metaplasia. The IR was 1.0 per 1000 person-years (95% confidence interval 0.7–1.3) among patients with non-dysplastic intestinal metaplasia, 0.9 (0.8–1.1) in non-dysplastic gastric/glandular metaplasia and 2.9 (2.0–4.2) among columnar metaplasia patients with low-grade dysplasia. The SIRs were 11.7 (95% confidence interval 8.6–15.5), 12.0 (10.0–14.2) and 30.2 (20.5–42.8), respectively. The SIRs for gastric cardia adenocarcinoma (GCA) were moderately elevated.

**Conclusions:**

For the first time, we demonstrate that patients with esophageal inflammatory and other metaplastic abnormalities than Barrett’s esophagus have an increased risk of EAC and GCA compared to the general population. Moreover, patients with different histopathologic subtypes of Barrett’s esophagus have a comparable risk for EAC.

**Supplementary Information:**

The online version contains supplementary material available at 10.1007/s10654-021-00833-6.

## Introduction

Gastroesophageal cancers including esophageal adenocarcinoma (EAC), esophageal squamous cell carcinoma (ESCC), gastric cardia adenocarcinoma (GCA) and gastric non-cardia adenocarcinoma (GNCA) are among the world’s most fatal cancers. Moreover, EAC and GCA are affecting increasingly more people in high-income countries [1, 2]. Correa’s cascade is a well described pathogenic pathway for GNCA and gives an excellent insight and target for surveillance and preventive treatment opportunities of precursor lesions in GNCA. Unfortunately, knowledge of the pathogenic pathways for EAC, ESCC and GCA is incomplete.

Barrett’s esophagus is the currently only known precursor lesion of EAC and GCA [3, 4]. Barrett’s esophagus is a condition where chronic exposure to gastric reflux changes or transforms the normal stratified squamous cell lining of the esophagus into metaplastic columnar epithelium, to better withstand the acidic gastric juice [1]. A growing body of observational studies has helped identify risk factors for malignant progression of Barrett’s esophagus such as segment length, presence of dysplasia and various demographic factors [5, 6]. Effective surveillance and treatment of Barrett’s esophagus may partly explain the severalfold lower annual risk for EAC (0.12–0.27%) from recent reports [7–14], compared with the previously reported mean annual risk estimates of 0.5% (range 0.1–3.5%) [8]. Moreover, only 5% of patients with incident EAC are found to have a prior diagnosis of Barrett’s esophagus [15]. Squamous dysplasia is currently the only known precursor lesion for ESCC [16].

There is an urgent need to better understand the pathogenic pathway leading to cancer development. Histopathological abnormalities in the esophagus in addition to Barrett’s esophagus and squamous dysplasia have been poorly explored this far. High-quality assessments of the malignant potential of other esophageal abnormalities are therefore needed.

Furthermore, a clarification is necessary of the specific risks of EAC associated with histopathologic subtypes of Barrett’s esophagus. A previous Danish study was based on histopathology reports, but did not separate intestinal metaplasia from gastric/glandular metaplasia in their report, possibly because of limited sample size [17], which requires further clarification since gastric metaplasia patients are not surveilled in many countries today.

We identified a large sample of patients with inflammatory and metaplastic abnormalities in the esophagus through Swedish histopathology registers. Our aim was to (1) explore the gastroesophageal cancer risk among patients with esophageal abnormalities and (2) estimate the risk for EAC by histopathologic subtypes of Barrett’s esophagus, since reports of this kind are rare in the previous literature.

## Materials and methods

### The research database

We used the ESPRESSO (Epidemiology Strengthened by histoPathology Reports in Sweden) study to construct a cohort of patients with esophageal biopsies, including those with Barrett’s esophagus.

Details of the ESPRESSO study have been reported previously [18]. In brief, the study population of 2.1 million individuals was constructed from 2015 to 2017 by retrieving information about date of biopsy, personal identity number, morphology and topography codes (according to the coding system SNOMED II) for biopsies taken between 1965 and 2017 from the gastrointestinal tract by all pathology departments in Sweden (n = 28). Using the personal identity number (PIN), a unique personal identity number assigned to each individual in Sweden [19], this study population was then linked to several nationwide registers to obtain background and healthcare information. Information about vital status (date of birth and death), age, sex, immigration, emigration, country of birth, education and income were delivered by Statistics Sweden, using data from the total population register [20] and the database LISA (longitudinal integrated database for health insurance and labor market studies) [21]. Healthcare data were retrieved from the following National Healthcare Registers maintained by the National Board of Health and Welfare: the Cause of Death Register [22] which contains > 99% of all deaths; the Swedish Cancer Register started in 1958 and covers > 96% of malignancies; the Patient Register including inpatient data since 1964, and hospital-based outpatient care since 2001, validated for usefulness in research for many diagnoses, but not for Barrett’s esophagus [23] and the Swedish Prescribed Drug Register with data from July 2005 and forward. The study was approved by the Regional Ethics Vetting Board of Stockholm (Swedish Ethical Review Authority) (Dnr 2014/1287-31/4 and 2020-00382).

### Sample selection

**Fig. 1:**
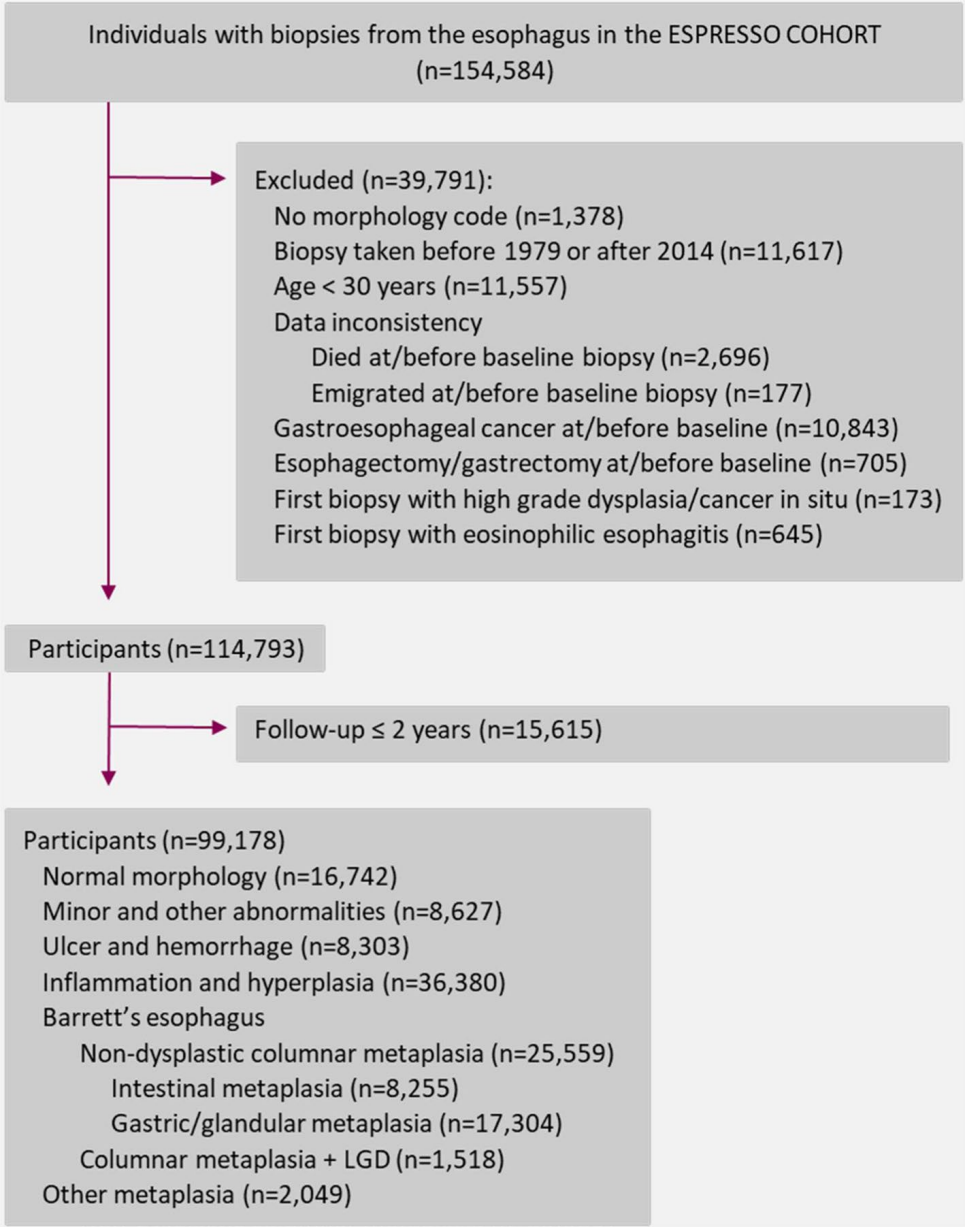
Sample selection of patients with esophageal biopsies from the ESPRESSO study in Sweden 1979–2014

A flowchart illustrating the sample selection from the ESPRESSO study is presented in Fig. 1. We identified 210,008 esophageal biopsies (topography code “T62”) from 154,584 unique individuals in the ESPRESSO study. The majority of the individuals had only been biopsied once, while 26% had been biopsied on multiple occasions, reaching up to 15 times. For each individual, we only kept the most severe biopsy finding from the first biopsy as baseline histopathology diagnosis. We excluded biopsies without a morphology code, those taken before 1979 (since large-scale registration of data in the pathology departments began in 1979) or after 2014 (2 years before the end of follow-up), patients younger than 30 years at first biopsy (since they might differ regarding etiology and risk according to previous literature [24–26]), individuals with data inconsistencies (died or emigrated at or before baseline biopsy), and patients with gastroesophageal cancer, esophagectomy or gastrectomy concurrent with or before the first biopsy. We also excluded patients with high-grade dysplasia, cancer in situ and eosinophilic esophagitis (Supplementary table 1) at the first biopsy since it was not within the scope of this study to examine these patients. Finally, 114,793 unique individuals remained in the study cohort. To reduce the risk of reverse causation, we further excluded the first two years of follow-up, resulting in 99,178 individuals to be kept for final analysis. We grouped the remaining esophageal biopsy findings according to morphology codes (Supplementary table 1) as normal morphology, minor and other mucosal abnormalities, ulcer and hemorrhage, inflammation and hyperplasia, non-dysplastic intestinal metaplasia, non-dysplastic gastric/glandular metaplasia, columnar metaplasia with low-grade dysplasia, and other metaplasia. We further categorized these eight groups into three biopsy groups according to morphology: normal (only including patients with normal morphology), inflammation (including minor and other mucosal abnormalities, ulcer and hemorrhage, inflammation and hyperplasia) and metaplasia (Barrett’s esophagus: non-dysplastic intestinal metaplasia, non-dysplastic gastric/glandular metaplasia, and columnar metaplasia with low-grade dysplasia; and other metaplasia). The current classification of groups of histopathological abnormalities was made according to neoplastic progression risk and not according to severity regarding needs for immediate clinical management.

### Statistical analysis

Characteristics of the participants are described with frequency, percent, median and interquartile range. Outcomes were defined as: EAC, ESCC, GCA or GNCA. Each participant was followed from two years after the date of the first biopsy to the diagnosis date of the outcomes or emigration, esophagectomy, gastrectomy, death or end-of-follow-up on the 31st of December 2016, whichever occurred first. We calculated incidence rates (per 1000 person-years) by dividing cases of gastroesophageal cancers with the number of the observed person-years, and calculated 95% confidence intervals (CIs) assuming a Poisson distribution of cases. We calculated standardized incidence ratios (SIRs) by dividing the observed number by the expected number of cancer cases, derived by multiplying sex-, age- (in 5-year intervals) and calendar year specific incidence rates for the general population with the person-years at risk accrued in our biopsy cohort. Test of trend was performed by a generalized linear model using Poisson distribution. Data on cancer incidence of the Swedish general population was acquired from the cancer statistics database at the National Board of Health and Welfare. Cumulative risk of developing gastroesophageal cancers two years after baseline diagnosis was estimated using the Nelson-Aalen method [27, 28].

**Fig. 2:**
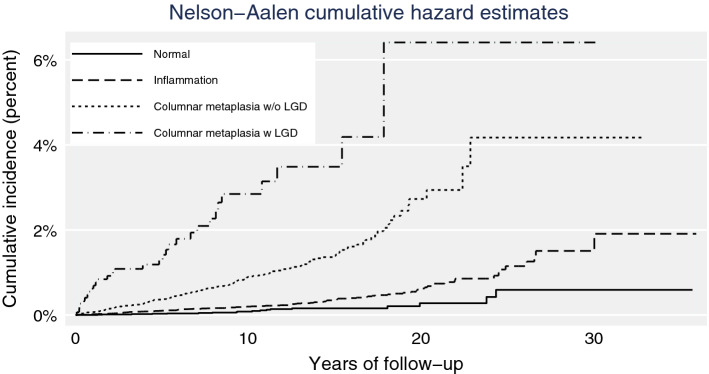
Cumulative incidence of EAC among esophageal biopsy patients in Sweden, 1979–2014, first two years of follow-up excluded. Inflammation includes minor abnormalities, other abnormalities, ulcer, hemorrhage, inflammation, hyperplasia; metaplasia without low-grade dysplasia includes other metaplasia and non-dysplastic intestinal/gastric/glandular metaplasia; columnar metaplasia with low-grade dysplasia includes columnar metaplasia with low-grade dysplasia

We used Cox proportional hazards regression to calculate hazard ratios (HRs) using attained age as the underlying time-scale. Here, occurrence of a more severe finding at biopsy was treated as a time-varying covariate and we adjusted for sex, birth cohort (before 1930 or 1930 and after), anti-reflux surgery as a time-varying covariate, education, weighted family income, country of birth, and diagnosis of alcoholism or chronic obstructive pulmonary disease (COPD) as time-varying proxy markers for heavy alcohol consumption and smoking. Since the Prescribed Drug Register started on 1 July 2005, in a sensitivity analysis further adjusting for drug use, we limited study subjects to those who underwent endoscopy after this date. We examined the proportional hazards assumption of the Cox model by using statistical tests based on Schoenfeld residuals. There was no indication of obvious violation of the proportional hazards assumption for any covariate. SAS software (version 9.4. Cary, NC, USA) was used for data extraction and statistical analyses. Figure 2 was produced using Stata software (Release 17. College Station, TX: StataCorp LLC).

## Results

### Patient cohort

Slightly more men (58%) than women had had an esophageal biopsy. The median age at the start of follow-up was 64 years and the biopsy cohort was followed for a median of 7 years (Table 1). The study cohort of 99,178 esophageal biopsy patients accrued 831,394 person-years during follow-up (Table 1). We further divided study subjects by histopathology into groups of normal morphology (N = 16,742), inflammation (N = 53,310) and metaplasia (N = 29,126). The group with normal esophageal morphology, compared with the other two groups had a more balanced distribution by sex and were younger at entry, and had longer median follow-up duration (Table 1).

**Table 1:** Characteristics of patients with esophageal biopsies in Sweden 1979–2014

Group by histopathology	No.	Men, n (%)	Median age at entry^a^ (IQR^b^)	Median follow-up, years (IQR^b^)	Person-years at risk
Total	99,178	57,116 (58)	64 (21)	7.2 (9.4)	831,394
Normal					
Normal morphology	16,742	8176 (49)	61 (21)	8.7 (10.2)	157,262
Inflammation					
Minor and other abnormalities	8627	4728 (55)	65 (21)	7.3 (10.0)	75,774
Ulcer and hemorrhage	8303	5110 (62)	70 (19)	6.2 (8.7)	64,661
Inflammation and hyperplasia	36,380	21,307 (59)	64 (21)	7.7 (9.9)	320,475
Metaplasia					
Barrett’s esophagus					
Non-dysplastic columnar metaplasia	25,559	15,534 (61)	64 (19)	6.3 (8.0)	183,406
Intestinal metaplasia	8255	5226 (63)	66 (18)	4.4 (6.2)	45,085
Gastric/glandular metaplasia	17,304	10,308 (60)	63 (19)	7.2 (8.3)	138,320
Columnar metaplasia + LGD^c^	1518	1048 (69)	67 (17)	5.8 (7.1)	10,547
Other metaplasia	2049	1213 (59)	64 (19)	8.5 (9.3)	19,269

^a^2 years after first biopsy

^b^Interquartile range

^c^Low-grade dysplasia

### Incidence rates, standardized incidence ratios, and cumulative risk for gastroesophageal cancers

The incidence rates ranged from 0.1 per 1000 person-years for those with normal morphology (annual risk 0.01%), to 2.9 per 1000 person-years for those with columnar metaplasia plus low-grade dysplasia (annual risk 0.29%). Compared to the general population, patients with normal morphology, minor and other abnormalities, ulcer and hemorrhage or inflammation and hyperplasia had two to six times increased risk for EAC (Table 2). As expected, subjects with more severe abnormalities had much higher excess risk for EAC. Among Barrett’s esophagus patients, those with non-dysplastic columnar metaplasia (both intestinal metaplasia and gastric/glandular metaplasia) had a 12-fold increased risk, while those with columnar metaplasia and low-grade dysplasia had about 30-fold increased risk, compared to the general population. Patients with other metaplasia also had a ten-fold increased risk (Table 2).

**Table 2:** Incidence rate (IR) and standardized incidence ratio (SIR) of gastroesophageal cancers among patients with esophageal biopsies in Sweden

Group by histopathology	Esophagus						Stomach					
	Adenocarcinoma			Squamous cell carcinoma			Cardia adenocarcinoma			Non-cardia adenocarcinoma		
	Obs	IR (1/1000 py)	SIR	Obs	IR (1/1000 py)	SIR	Obs	IR (1/1000 py)	SIR	Obs	IR (1/1000 py)	SIR
Normal morphology	18	0.1	1.8 (1.1,2.9)	14	0.1	1.6 (0.9,2.7)	10	0.1	1.1 (0.5,1.9)	23	0.1	0.8 (0.5,1.2)
Inflammation												
Minor/other	28	0.4	5.1 (3.4,7.4)	16	0.2	3.3 (1.9,5.3)	17	0.2	3.1 (1.8,5.0)	10	0.1	0.6 (0.3,1.1)
Ulcer/hemorrhage	32	0.5	5.7 (3.9,8.1)	4	0.1	0.8 (0.2,2.0)	13	0.2	2.3 (1.2,3.9)	9	0.1	0.5 (0.2,0.9)
Inflammation/hyperplasia	64	0.2	2.7 (2.1,3.4)	41	0.1	2.1 (1.5,2.8)	29	0.1	1.3 (0.9,1.9)	49	0.1	0.7 (0.5,1.0)
Metaplasia												
Barrett’s esophagus												
Non-dysplastic columnar metaplasia	179	0.9	11.9 (10.2,13.8)	13	0.1	1.2 (0.6,2.0)	49	0.3	3.7 (2.8,4.9)	27	0.1	0.8 (0.5,1.2)
Intestinal metaplasia	47	1.0	11.7 (8.6,15.5)	3	0.1	1.1 (0.2,3.2)	12	0.3	3.6 (1.9,6.3)	8	0.2	1.0 (0.5,2.1)
Gastric/glandular metaplasia	132	0.9	12.0 (10.0,14.2)	10	0.1	1.2 (0.6,2.2)	37	0.3	3.8 (2.7,5.2)	19	0.1	0.7 (0.4,1.2)
Columnar metaplasia + LGD	31	2.9	30.2 (20.5,42.8)	2	0.2	2.8 (0.3,10.2)	12	1.1	13.7 (7.1,24.0)	2	0.2	0.9 (0.1,3.4)
Other metaplasia	15	0.8	9.7 (5.4,15.9)	2	0.1	1.6 (0.2,5.9)	3	0.2	2.1 (0.4,6.1)	5	0.3	1.2 (0.4,2.8)

Obs, observed number of cancer cases; py, person-years

Figure 2 shows the cumulative incidence for EAC among patients grouped by severity of histopathological abnormalities. The slope of the cumulative risk curves showed great difference for the 4 groups, with the steepest one observed for those with columnar metaplasia plus low-grade dysplasia. Up to 20 years of follow-up, the cumulative incidence was 0.28% for the normal group, 0.62% for those with inflammation, 2.73% for those with metaplasia without low-grade dysplasia, and 6.41% for those with columnar metaplasia and low-grade dysplasia.

Patients with esophageal abnormalities showed moderately but statistically significant elevated risks for ESCC, i.e., SIR 3.3 (95% CI 1.9–5.3) among patients with minor/other abnormalities (including squamous high-grade dysplasia) and SIR 2.1 (95% CI 1.5–2.8) among inflammation/hyperplasia patients. Except for patients with a normal morphology, inflammation/hyperplasia and other metaplasia, patients with other abnormalities had shown elevated risks to develop GCA compared to the general population, but not in the same magnitude as EAC (SIRs ranged from 2.3 for those with ulcer/hemorrhage to 13.7 for those with columnar metaplasia plus low-grade dysplasia). None of the groups experienced an increased risk for GNCA (Table 2).

### Standardized incidence ratios for gastroesophageal adenocarcinoma among patients with non-dysplastic columnar metaplasia, stratified by sex, age, follow-up duration and calendar year

Among patients with non-dysplastic columnar metaplasia, although men had a higher incidence rate for EAC than women, no obvious difference of relative risk was seen according to sex (SIR 11.8 vs 12.6, for men and women, respectively). Furthermore, with increasing age at entry, the incidence rates for EAC increased, while the relative risks compared to the general population decreased (SIR 64.2 vs 9.8 for age at entry of 30–49 and ≥ 70, respectively). Moreover, there was a trend of decreasing relative risks for EAC with longer duration of follow-up (P_trend_ < 0.001), and more recent calendar period at entry, but the latter was not statistically significant. The pattern of SIRs was similar for GCA but with a lower magnitude (Table 3).

**Table 3:** Incidence rate (IR) and standardized incidence ratio (SIR) of esophageal or cardia adenocarcinoma among patients with non-dysplastic columnar metaplasia according to sex, age at entry, follow-up duration and calendar year at entry

Characteristics	Esophageal adenocarcinoma			Gastric cardia adenocarcinoma		
	Obs	IR (1/1000 py) (95% CI)	SIR (95% CI)	Obs	IR (1/1000 py) (95%CI)	SIR (95% CI)
Sex						
Men	157	1.4 (1.2,1.6)	11.8 (10.0,13.8)	43	0.4 (0.3,0.5)	3.9 (2.8,5.2)
Women	22	0.3 (0.2,0.5)	12.6 (7.9,19.1)	6	0.1 (0.0,0.2)	3.0 (1.1,6.5)
Age group at entry						
30–49 years	8	0.4 (0.2,0.7)	64.2 (27.7,126.4)	2	0.1 (0.0,0.3)	11.6 (1.4,42.1)
50–59 years	22	0.6 (0.4,1.0)	20.2 (12.7,30.6)	7	0.2 (0.1,0.4)	6.6 (2.6,13.6)
60–69 years	55	1.0 (0.8,1.3)	12.8 (9.6,16.6)	19	0.4 (0.2,0.6)	5.2 (3.1,8.1)
≥ 70 years	94	1.2 (1.0,1.5)	9.8 (8.0,12.1)	21	0.3 (0.2,0.4)	2.6 (1.6,3.9)
* P* for trend			< .0001			< .0001
Follow-up duration, years						
0 to < 5	86	2.9 (2.3,3.6)	32.5 (26.0,40.2)	24	0.8 (0.5,1.2)	10.6 (6.8,15.7)
5 to < 10	56	1.0 (0.7,1.3)	11.9 (9.0,15.5)	15	0.3 (0.1,0.4)	3.7 (2.1,6.1)
10 to < 15	21	0.3 (0.2,0.5)	4.6 (2.9,7.1)	7	0.1 (0.0,0.2)	1.8 (0.7,3.6)
≥ 15	16	0.4 (0.2,0.6)	5.1 (2.9,8.2)	3	0.1 (0.0,0.2)	1.1 (0.2,3.1)
* P* for trend			< .0001			< .0001
Calendar year at entry						
1981–1999	67	1.8 (1.4,2.2)	21.9 (17.0,27.8)	16	0.4 (0.2,0.7)	5.2 (2.9,8.4)
2000–2006	74	0.9 (0.7,1.1)	11.1 (8.7,13.9)	18	0.2 (0.1,0.3)	3.1 (1.9,5.0)
2007–2016	38	0.6 (0.4,0.8)	7.1 (5.1,9.8)	15	0.2 (0.1,0.4)	3.5 (1.9, 5.7)
* P* for trend			0.4762			0.6403

Obs, observed number of cancer cases; py, person-years

### Analysis of risk of esophageal and cardia adenocarcinoma among esophageal biopsy patients by taking into account follow-up biopsy

Finally, we included findings during follow-up biopsies as a time-varying variable in the calculation of SIR and found that the SIR for EAC increased incrementally from the group with normal morphology (SIR 1.5; 95% CI 0.8–2.6) to inflammation (3.1; 95% CI 2.6–3.8), metaplasia without low-grade dysplasia (11.1; 95% CI 9.6–12.8), and columnar metaplasia with low-grade dysplasia (24.9; 95% CI 18.7–32.6), a trend which was also seen for GCA but with a lower magnitude (Table 4). In the corresponding Cox model with full adjustment for potential confounders, compared to those with normal morphology, those with inflammation, metaplasia without low-grade dysplasia, and columnar metaplasia plus low-grade dysplasia had around twofold (95% CI 1.2–3.5), eightfold (95% CI 4.9–14.6) and 20-fold (95% CI 11.1–36.2) increased hazards to develop EAC (Table 4). A similar trend was also found for GCA although with a lower magnitude. The sensitivity analysis restricting study subjects to patients who underwent esophageal biopsy after 1st of July 2005, and further adjusting for drug treatments (proton pump inhibitor, H2 inhibitor, non-steroidal anti-inflammatory drug and *Helicobacter pylori* eradication therapy), showed similar results for EAC and GCA, respectively (Supplementary table 2).

**Table 4:** Standardized incidence ratio (SIR), hazard ratio (HR), and their 95% confidence intervals (CIs) for esophageal adenocarcinoma and gastric cardia adenocarcinoma among patients with esophageal biopsies, by taking into account progression of histopathological group during follow-up

Characteristics	Esophageal adenocarcinoma				Gastric cardia adenocarcinoma			
	Cases	SIR (95% CI)	HR^a^ (95% CI)	*P* value	Cases	SIR (95% CI)	HR^a^ (95% CI)	*P* value
Group by histopathology^b^								
Normal	14	1.5 (0.8,2.6)	Ref		9	1.0 (0.4,1.9)	Ref	
Inflammation^c^	105	3.1 (2.6,3.8)	2.0 (1.2,3.5)	0.0129	58	1.7 (1.3,2.2)	1.8 (0.9,3.7)	0.0945
Metaplasia without LGD	195	11.1 (9.6,12.8)	8.4 (4.9,14.6)	< .0001	51	2.9 (2.2,3.8)	3.5 (1.7,7.2)	0.0005
Columnar metaplasia with LGD	53	24.9 (18.7,32.6)	20.0 (11.1,36.2)	< .0001	15	7.0 (3.9,11.6)	9.4 (4.1,21.6)	< .0001

^a^Attained age as time scale, and adjusted for sex, birth cohort, anti-reflux surgery (time-varying), education, weighted family income, country of birth, alcoholism (time-varying), and COPD (time-varying)

^b^Treated as time-varying variable. Changes 2 years before cancer occurrence were disregarded. Inflammation includes minor abnormalities, other abnormalities, ulcer, hemorrhage, inflammation, hyperplasia; metaplasia without low-grade dysplasia includes other metaplasia, non-dysplastic intestinal/gastric/glandular metaplasia; columnar metaplasia with low-grade dysplasia includes columnar metaplasia with low-grade dysplasia

^c^Among whom 2030 patients were diagnosed to have metaplasia during follow-up endoscopies

## Discussion

In our study, the risk for EAC was two to five times higher among patients with inflammatory histopathologic abnormalities, twelve times higher in non-dysplastic intestinal metaplasia patients and gastric/glandular metaplasia patients, and thirty times higher among columnar metaplasia patients with low-grade dysplasia, compared to the general population. Our estimate is in line with a similar histopathology-register-based study in Denmark [7], and pooled estimates from previous meta-analyses [14, 29–31]. However, our study is the first to analyze inflammatory abnormalities and furthermore, intestinal and gastric/glandular metaplasia patients separately and demonstrated a similar risk in these two groups. We also demonstrated a 9.7-fold elevated risk for EAC among patients with other metaplasia types comparable to non-dysplastic Barrett’s esophagus. These findings implicate that patients with inflammatory abnormalities and non-dysplastic metaplasia may continue current surveillance and/or treatment procedures in Sweden, while those with dysplastic changes could benefit from strengthened surveillance.

This is the largest histopathology-register-based study of esophageal abnormalities to this date with linkage to nearly complete nationwide registers with high-quality data which decreases the risk of selection bias due to loss of follow-up. The histopathology registers are based on a nearly free-of-charge universal health-care system, with a decreased risk for referral bias.

Among patients with non-dysplastic columnar metaplasia, men experienced higher incidence rate for EAC compared to women, which is in line with previous assessments [32]. The SIR, being a relative risk with the corresponding sex-specific general population as reference group, was not markedly different between the sexes, as expected. The remarkably high SIR estimate in patients under the age of 50 has not been reported previously and is due to very low EAC incidence in the general population under 50 years of age. It indicates that EAC occurs earlier in Barrett’s esophagus patients, which may be partly due to detection bias, or that Barrett’s esophagus is developing in younger populations. However, concerns have been raised in current literature that Barrett’s esophagus is increasingly diagnosed in younger patients, but this does not seem to translate into an increase in EAC in young adults [33]. We also found that non-dysplastic columnar metaplasia patients who had their first biopsy taken 2007–2016 had lowest relative risk for EAC compared to patients enrolled earlier. This may be explained by the relatively short follow-up time, and/or better management of this patient-group by the health-care system than previously. Reporting practices in pathology departments and endoscopic surveillance have changed considerably over the years, thus emphasis should be on the most recent 2007–2016 data, which is most relevant to today's clinical practices. Low-grade dysplasia patients had the most elevated risk for EAC in the group with severe esophageal abnormalities. The accurate diagnosis rate of low-grade dysplasia is closely linked to the estimated risk for progression and if it is low in the current cohort, this together with introduced more rigorous treatment may explain the lower incidence (annual risk 0.3% (0.2–0.4%)) for EAC among columnar metaplasia patients with low-grade dysplasia in our dataset compared to previous estimates. Furthermore, expert histological review of low-grade dysplasia in patients with Barrett’s esophagus is part of current clinical practice whereby the majority of cases are downstaged and have a low risk of progression, while in a minority of patients with confirmed low-grade dysplasia there is a markedly increased risk of malignant progression [34]. Here, information about expert histological review is lacking but is most likely only performed in a minority of low-grade dysplasia patients, which could further contribute to the lower incidence for EAC among patients with low-grade dysplasia.

Sampling, coding and diagnosis error might contribute to misclassification of non-dysplastic intestinal metaplasia as gastric/glandular metaplasia and vice versa, which can partly explain the observed similar risks for EAC between these groups.

Patients with other metaplasia types (mainly consisting of metaplasia ‘not otherwise specified’) displayed an increased risk for EAC which may be due to misclassification of the diagnosis and could be indicative of the histopathology diagnostic bias. The observed increased risk for EAC among patients with other metaplasia types is likely to reflect either that other metaplasia types contribute to the development of Barrett’s esophagus and then EAC or that Barrett’s esophagus is prone to be misdiagnosed in the presence of other metaplasia types so that it was present but not sampled, or not seen at endoscopy or pathological review due to the inflammatory environment. A previous study reported that ulceration present in the context of Barrett’s esophagus increased the risk of EAC [35]. The risk for GCA was increased in a similar manner as for EAC but not as prominent among all esophageal biopsy groups, although the excess risk was not statistically significant for normal morphology, inflammation/hyperplasia and other metaplasia, possibly reflecting a partly shared etiological pathway. The risk for ESCC was increased among patients with minor/other abnormalities and inflammation/hyperplasia, possibly harboring squamous pre-neoplastic abnormalities. None of the esophageal biopsy groups was associated with an increased risk for GNCA, which was expected considering no such associations have been reported in the previous literature.

Barrett’s esophagus alone could explain the EAC “cancer epidemic” during the last decades. Computational modelling suggests that there are likely few cases of EAC outside of the expected cases from Barrett’s esophagus [36]. What is the clinical value of identifying other inflammatory and metaplastic mucosal changes that are associated with an increased risk for EAC? The majority of patients with Barrett’s esophagus do not develop EAC, which is why screening and surveillance of Barrett’s esophagus is recommended against in most countries. Hence, most of the patients with Barrett’s esophagus destined to develop EAC could be captured with effective surveillance of high-risk individuals, but will likely remain undiagnosed until a less invasive and cost-effective diagnostic and prognostic tool than endoscopy emerges for Barrett’s esophagus in clinical practice [37]. Here, we have identified that inflammatory changes and other metaplasia types than intestinal metaplasia, most likely contribute to the development of Barrett’s esophagus or increase the risk of misdiagnosis of Barrett’s esophagus. Furthermore, the scientific understanding of the cellular origin of Barrett’s esophagus is incomplete [4]. Until the cellular pathophysiology has been mapped, there is still a risk that additional pathways leading to gastroesophageal cancer development have been overlooked. An example of this is a minor pathway to EAC other than the established flow from GERD through Barrett’s esophagus to dysplasia and later EAC [38] where primary adenocarcinoma cases arise from ectopic gastric mucosa, so called “inlet patch” in the upper esophagus noted in several case reports [5, 38–43].

Limitations of our study are the lack of information from the endoscopic examination about the reason for referral to endoscopy, location of the biopsy and segment length. Lead time until biopsy from the underlying disease is also unknown. Since esophageal cancer symptoms typically present at an advanced disease stage, the progression risk among asymptomatic patients are difficult to estimate. Esophageal biopsies are not routinely performed on patients undergoing endoscopy. The clinical indication for an esophageal biopsy might thus be a risk factor for the development of EAC. The registration of high-grade dysplasia is of low-quality since it is not covered by the Swedish Cancer Register which is why we did not include high-grade dysplasia as an end-point. The coverage of patients with high-grade dysplasia in our database is incomplete and could not serve as reliable outcome marker. Our risk estimates are therefore comparable to the single outcome of EAC, but lower than those for the joint outcome combining high grade dysplasia/EAC. The ESPRESSO cohort does not include undiagnosed Barrett’s esophagus patients in Sweden, as a previous population-based study estimated the prevalence of Barrett’s esophagus in 1.6% of the general Swedish population [44]. The true risk might be lower than current estimates, as this patient cohort could be skewed due to the requirement of a clinically indicated endoscopy and biopsy for various indications. We were unable to assess the prevalence of Barrett’s esophagus by a follow-up endoscopy among patients after a resolved inflammation. This could have led to an overestimation of the risk of neoplastic progression in patients with esophagitis. Detection and diagnostic bias is also inherent when data from routine clinic histopathology registers are used which may explain observed differences in the risk for EAC. Finally, we cannot rule out some heterogeneity in the SNOMED use between pathologists and/or Swedish pathology departments.

We conclude that patients with inflammatory abnormalities have a moderately increased risk and patients with Barrett’s esophagus have a lower risk for esophageal adenocarcinoma than previously reported. Among patients with non-dysplastic columnar metaplasia, the risk for EAC was similar irrespective of histopathological subtypes. Patients with other metaplasia types also have a significantly increased risk of EAC which requires further investigation.

## Supplementary Information

Below is the link to the electronic supplementary material.

Supplementary file1 (DOCX 38 KB)

## Abbreviations

EAC: Esophageal adenocarcinoma
ESCC: Esophageal squamous cell carcinoma
GCA: Gastric cardia adenocarcinoma
GNCA: Gastric non-cardia adenocarcinoma
