# Supplementary material for: Esophageal abnormalities and the risk for gastroesophageal cancers—a histopathology-register-based study in Sweden
Source: Eur J Epidemiol. 2022 Jan 3;37(4):401–11. doi: 10.1007/s10654-021-00833-6 (PMC9187549; doi:10.1007/s10654-021-00833-6)
Supplement: Supplementary file 1 — Supplementary file1 (DOCX 38 KB) [file 10654_2021_833_MOESM1_ESM.docx]

**Supplementary figure 1.** Incidence rate (1/1000 person-years) for EAC among Barrett’s esophagus patients in the ESPRESSO cohort (non-dysplastic intestinal metaplasia=NDIM, non-dysplastic gastric/glandular metaplasia=NDGM, columnar metaplasia with low-grade dysplasia=LDCM), Sweden, 1979-2014, compared to incidence rates from previous published studies among patients with non-dysplastic Barrett’s esophagus (NDBE), Barrett’s esophagus with low-grade dysplasia (LDBE), Barrett’s esophagus (BE), short-segment Barrett’s esophagus (SSBE) and long-segment Barrett’s esophagus (LSBE).

**Supplementary table 1.** Codes used in the study

| Source/system | Label | Code |
| --- | --- | --- |
| Cancer register:  ICD7 and PAD | esophageal adenocarcinoma  esophageal squamous cell carcinoma  cardia adenocarcinoma  non-cardia adenocarcinoma  other esophageal or gastric cancer | 150 + 096  150 + 146  1511 + 096  151 (not 1511) + 096  150 + 151 (not 096 or 146) |
| Pathology register:  SNOMED II | normal  minor and  other changes (necrosis, atrophy, hypertrophy, proliferation, polyp, H. pylori, sq atypia, sq dysplasia)  hemorrhage and ulcer  inflammation and hyperplasia  other metaplasia (NOS, squamous, osseous, myeloid, cystitis cystica)  columnar metaplasia (intestinal\|gastric\|glandular)  gastric and/or glandular metaplasia  intestinal metaplasia  columnar metaplasia and low-grade dysplasia  high-grade dysplasia or adenocarcinoma in situ  eosinophilic esophagitis | M001  M0 (not M001), M1 (not M14110), M2, M3 (not M37000,M38),  M5, M6 (not M697), M7 (not M72), M8 (not M80702), M9  M37000,M38  M4, M72, M14110  M73220, M73200, M73225, M73000, M73400, M73370, M73500  M73320\| M73330, M73335 \| M73300, M73301, M73305, M73309  M73330, M73335 \| M73300, M73301, M73305, M73309  M73320  columnar metaplasia as above\|M73325\| M69726 \| M69727  M69728 + M69729 \| M81402  M47150 |
| Patient register:  Klassifikation av operationer and ICD | Esophagectomy  Gastrectomy  Anti-reflux surgery  Alcoholism  COPD | 2820\|2821\|2822\|2829\|JCC00\|JCC10\|JCC11\|JCC20\|JCC30\|JCC96\|JCC97  4411\|4412\|4413\|4414\|4415\|4416\|4417\|4418\|4419\|4420\|4421\|4422\|  4423\|4424\|4425\|4426\|4429\|JDC00\|JDC10\|JDC11\|JDC20\|JDC30\|JDC40\|JDC96\|JDC97  4270\|4271\|4272\|JBA\|JBB\|JBC\|JBW  1964-1968: 322.XX, 307.XX, 364.XX, 431.XX, 540-545.XX, 581.XX, 587.XX  1969-1986: 291.XX, 303.XX, 577.10, 571.09, 571.10  1987-1996: 291.X, 303.X, 305.X, 535.X, 571.X, 357.X, 425.X, V79.X  1997-: F10.X (ej F100), G31.2, G62.1, G72.1, I42.6, K29.2, K86.0, Z72.1  1964-1968: 500.XX, 501.XX, 502.XX  1969-1986: 491.XX, 492.XX  1987-1996: 490-496.X  1997-: J41.X, J42.X, J43.X, J44.X |
| Prescribed drug register:  ATC | PPI  H2-inhibitor  NSAID  *Helicobacter pylori* eradication | A02BC  A02BA  M01A  A02BD06 (J01CA04, J01FA09, J01XD01) |

**Supplementary table 2.** Sensitivity analysis of standardized incidence ratio (SIR), hazard ratio (HR), and their 95% confidence intervals (CIs) for esophageal adenocarcinoma and gastric cardia adenocarcinoma among patients with esophageal biopsies during 2005-2014, by taking into account progression of histopathological group during follow-up, adjusting for drug treatments.

|  | Characteristics |  | Esophageal adenocarcinoma | | |  | Gastric cardia adenocarcinoma | | |  |
| --- | --- | --- | --- | --- | --- | --- | --- | --- | --- | --- |
|  |  |  | Cases | SIR (95% CI) | HR^a^ (95% CI) |  | Cases | SIR (95% CI) | HR^a^ (95% CI) |  |
| **Group by histopathology**^b^ | |  |  |  |  |  |  |  |  |  |
| Normal | |  | 3 | 1.3 (0.3,3.7) | Ref | *P*-value | 1 | 0.4 (0.0,2.4) | Ref | *P*-value |
| Inflammation | |  | 12 | 1.2 (0.6,2.2) | 0.9 (0.3,3.2) | 0.8684 | 19 | 2.0 (1.2,3.1) | 4.7 (0.6,35.1) | 0.1328 |
| Metaplasia without LGD | |  | 50 | 7.0 (5.2,9.3) | 5.3 (1.6,16.9) | 0.0055 | 20 | 2.8 (1.7,4.3) | 6.1 (0.8,46.0) | 0.0770 |
| Columnar metaplasia w LGD | |  | 16 | 22.8 (13.0,37.0) | 17.8 (5.1,61.8) | <.0001 | 7 | 10.0 (4.0,20.5) | 21.8 (2.7,179.0) | 0.0041 |

^a^ Attained age as time scale, and adjusted for sex, birth cohort, anti-reflux surgery (time-varying), education, weighted family income, country of birth, alcoholism (time-varying), COPD (time-varying) and drug treatments (proton pump inhibitor, H2 inhibitor, non-steroidal anti-inflammatory drug and Helicobacter pylori eradication therapy).
^b^ Treated as time-varying variable. Changes 2 years before cancer occurrence were disregarded. Inflammation include minor abnormalities, other abnormalities, ulcer, hemorrhage, inflammation, hyperplasia; metaplasia without low-grade dysplasia include other metaplasia, non-dysplastic intestinal/gastric/glandular metaplasia; columnar metaplasia with low-grade dysplasia includes columnar metaplasia with low-grade dysplasia.
